# Major Extremity Injuries Associated with Farmyard Accidents

**DOI:** 10.1100/2012/314038

**Published:** 2012-04-26

**Authors:** Cem Copuroglu, Nurettin Heybeli, Mert Ozcan, Baris Yilmaz, Mert Ciftdemir, Elif Copuroglu

**Affiliations:** ^1^Department of Orthopaedics and Traumatology, Trakya University, 22050 Edirne, Turkey; ^2^Department of Anesthesiology, Trakya University, 22050 Edirne, Turkey

## Abstract

*Background*. The aim of the study is to analyze the major agricultural injuries related to the extremities. *Patients*. We evaluated a 3-year period including 41 patients. Data on age, sex, injury patterns, anatomical localizations, injury season, length of stay in the hospital, and infections were evaluated, and the patients were examined with SF-36 in the follow-up period. *Results*. Hand was the most commonly injured part (*n*: 9) followed by the distal part of the lower limb (cruris) (*n*: 7) and foot (*n*: 7). Mean time between trauma and emergency-department arrival was 115 minutes (60–360). Mean length of stay was 24 days (4–150), and mean number of operations during hospitalization was 2.4 (1–30). Deep wound infection was seen in 8 patients. Seasonal distribution for accidents was even for spring and fall (27% each), high for summer (36%), and less for winter (10%). *Conclusions*. Distal parts of the elbow and knee were affected more frequently. Due to the high microbiological load and high incidence of crush-type injuries, repetitive debridements and long duration of hospital stay were needed. Attention should be paid in the harvesting times to the farmyard injuries. Due to the seasonal variation, more resources should be allocated to treat the increasing incidence of injury over the period from spring to fall.

## 1. Introduction

Agricultural work-related injury can be defined as an acute injury that occurs while doing farm work or travelling to or from work [1]. Farming as an occupation demands on many factors which include perception skills as well as the ability to perform complex and repetitive tasks, and it is one of the most hazardous vocations [2]. The integration of these skills influences a persons' ability to work safely in the farm workplace, and deterioration of any of these skills may increase the risk of injury [3]. There is a great variety of farm accidents, and this spectrum of injuries consists of simple lacerations to traumatic amputations. Farming accidents do not only depend on human factors but also environmental and machinery factors [4–6]. 

Farm injuries are important causes of mortality and morbidity for all age groups. Farmyard injuries result in major physical and psychological impacts as well as economical burdens. Economic costs have been studied in the USA, and agricultural occupational injuries cost $4.57 billion (range $3.14 billion to $13.99 billion) in 1992. When evaluated on per person, farming accidents cost 30% more than the national average to occupational injuries [7].

Despite the fact that our region is one of the major farming areas of our country, the data on agricultural injury extent is sparse. In order to establish preventive measures and policies, research should be done on demographical properties, injury types, and related morbidities. Harvest times are hardworking times of the farmers so that they are prone to farmyard injuries. This may be the result of the work done at harvest time which is more dangerous than at any other time due to the work pattern and machinery involved. Therefore, in this study, we aimed to focus on (1) the types and incidences of extremity injuries with an emphasis on seasonal variation, (2) the severity of these injuries, (3) description of the treatment required especially for infection and possibility of amputation, and (4) outcome of treatment and successful return to work or resumption of prior work capacity.

## 2. Patients and Methods

This is a retrospective case series study, and data were collected from hospitalized farm injury patients over a three-year period (between October, 2005 and October, 2008). The patients were treated in our University Hospital Orthopaedics and Traumatology Department which accepts patients whose treatment could not be completed at smaller hospitals of our region. Age, gender, trauma type and anatomical localizations, date of the trauma in order to object whether there is seasonal dispersion pattern, time to reach to hospital, treatment details including microbiology, and hospitalization period were evaluated according to the existing data. The main inclusion criterion of the study group was extremity trauma necessitating operative intervention, which has happened in the farmyard. Isolated skull, maxillofacial, spine, thorax and abdominal injuries were excluded from the study as well as farm animal bites, agricultural chemicals, and dust and airborne toxin hazards. Because of our hospital being a referral center, our study group included a high percentage of open fractures. The protocols for our approach to open fractures were as follows. First step of the management of open injuries included extensive debridement and irrigation. During the first intervention, under general anesthesia, the wound is irrigated and microbiology swabs are taken, then following this, the wound is debrided. After debridement of the wound, the fracture is temporarily fixed with a spanning external fixator. After culture specimens are taken, wide spectrum antibiotherapy (1st-generation sefalosporin, gentamycin and aminoglikozid for anaerobe microorganisms) and tetanus prophylaxis and applied. During the hospitalization period all the open wounds are redebrided and new specimens (deep tissue biopsies) are collected in the operating room and these specimens are incubated in the Microbiology Department. According to these culture antibiograms, depending on the microorganism, we use medical antibiotherapy for soft tissue and bone infection. During this period, repetitive debridements are applied, and after being sure that there is no microorganism in the deep tissue cultures, and ability of wound closure, we use ultimate fixation techniques for the fractures and then close the wound. The patients were called for followup to the outpatient clinic in March, 2010. Ethical review process was not a must during the study period, for such a retrospective case series study in our country, so the study does not have any ethics committee approval.

## 3. Results

There were 41 patients, 40 males and one female; the mean age was 42 (range 11 to 75).

### 3.1. Types of Injuries

**Figure 1:**
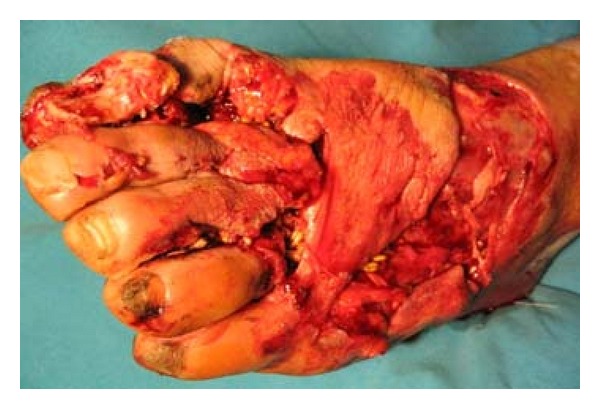
Foot crush injury in a farmyard accident. Wheat can be seen in the injured foot.

Forty of the patients had bone fractures, and 1 had soft-tissue laceration around the knee. Eleven of these fractures were crush injuries. Twenty three of the fractures were open fractures (13 Grade 3, 4 Grade 2, and 6 Grade 1 according to Gustilo-Andersson Classification). One of the open injuries can be seen in Figure 1.

Thirty-four of total cases had multiple bone fractures, and 6 had single bone fracture. Two patients had fractures of different extremities concomitantly. Hand was the most commonly injured part followed by cruris and foot (Table 1).

The fractures in the study group were fixed with external fixation, percutaneous pinning, intramedullary nailing, and open reduction and internal fixation techniques. One cruris injured patient had nonunion in the follow-up period and reoperated for pseudoarthrosis. Injury mechanisms were mostly high-energy injuries. The most common etiologic factor for an extremity injury was a farm machine injury followed by a tractor-dependent injury (Table 2).

Seasonal distribution for accidents was even for spring and fall, while it was found relatively high for summer and relatively less for winter (Table 3).

Mean application time of the patients to our Emergency Department after the trauma was 115 minutes (range 60 to 360 minutes). Mean hospitalization time for the patients was 24 days (range 4 to 150 days). Twenty-one cases applied directly to our hospital, and 20 cases were referred from other trauma centers. Mean time of first intervention was 1.4 hours in the directly applied patients. Three of the patients were referred to our hospital from our city, and mean first intervention time was 1.7 hours, 12 patients were referred from other trauma centers closer than 60 kilometers and mean first intervention time was 4.1 hours, and 5 patients were referred from longer distances (more than 60 km), and mean intervention time was 4.7 hours.

### 3.2. Severity of the Injuries:

Twenty-three of the 41 patients had soft-tissue injuries which need intervention. In 10 patients, skin defects were covered with split-thickness skin grafts, and the functional results were good. In 2 cases with complex soft-tissue injuries, skin defects were covered with flaps. The other open wounds healed by secondary wound healing. Three patients had flexor tendon repair. Six patients had vascular injuries, two of them necessitating vascular surgical interventions. One of the patients had skull injury, and one had abdominal injury concomitant to the extremity injuries.

### 3.3. Infection/Amputation:

In 8 of 41 patients deep wound infections were noticed. All were treated with repetitive debridement and antibiotic therapy. In deep tissue cultures, 3 acinetobacter, 3 enterobacteriaceae, 1 methicillin resistant staphylococcus aureus and 1 Klebsiella with enterobacteriaceae specimens were incubated. One acinetobacter and 1 enterobacteria-infected cases went to above-knee amputation because of the continuous infection. One ankle-fractured patient was followed for osteomyelitis in the follow-up period. Details about the patients with deep wound infections were shown in Table 4. There were five traumatic amputations (1 forearm, 3 above elbow, and 1 below knee) one delayed amputation due to failure of vascular repair. Because of the extensive soft-tissue damage and necrosis, it had to be amputated at day 6. Three late amputations had to be performed on 3 patients, two of them above the knee and one above the elbow because of severe infection and soft-tissue problems. Amputated patients returned to work but were disabled (had to perform at a lower level of function).

Mean follow-up time was 46 months (range 17 to 89 months). Thirty-six patients out of 41 were examined. Four patients (one hand injured three lower extremity injured) could not be reached, and one patient (multiextremity injured) was dead because of a heart disease during the follow-up period, so five of the 41 patients could not be evaluated. According to physical function, lower-extremity-injured patients had worse results. When all the patients are evaluated, 31 had good results. When pain was taken into consideration, upper-extremity-injured patients complained more, and 30 of all patients had good results. According to physical role difficulty, no significant differences could be obtained, and 22 had good results. According to general health status, 27 had good results and upper-extremity-injured patients had better results. According to vitality, 29 had good results and lower-extremity-injured patients had better results. According to social function, 31 of the patients had good results, no significant difference according to the injury localization could be obtained. Emotional role difficulty results were better in the lower-extremity-injured patients and 22 had good results. Mental health evaluation was better in the lower-extremity-injured patients and 31 had good results. By the final evaluation, depending on the social function and physical role difficulty, and no significant differences could be obtained related to the injury localization. Depending on the physical function and general health, upper extremity injured patients had better results. When pain, vitality (energy), emotional role difficulty and mental health were evaluated, lower-extremity-injured patients had better results. 

## 4. Discussion 

Farm injuries are important causes of mortality and morbidity [5] and have higher rates of occupational accidents than most industries [8]. Most of the injuries are based on machinery accidents, so they are mostly high energy injuries which are prone to serious complications and high economic costs. People employed in the farming industry often work in isolation, thus first aid or medical assistance may be delayed for farmyard injuries. Transport to the nearest medical center may be time consuming as well. Such reasons make farm injuries challenging and important so as to be prevented for all age groups. In this study, we aimed to define the characteristics and associated morbidity of farmyard injury to extremities. There are not many studies which are based on farmyard injuries from our country although 40% of the population is living at rural areas [9]. However, our study has faced certain limitations. Being a retrospective review, a longitudinal approach to patients' health conditions could not be focused. Another limitation was our hospitals being a university clinic; our series included cases that do not represent a general profile for farmyard injury. This can explain the high percentage of open fractures (56.1%) in this series which probably do not represent a general data. 

In some study groups, leading mechanism of accidents for nonfatal farmyard injuries were machinery accidents [6, 10–12]. In a study of 8129 male farmers aged 66 and older, Voaklander et al. [3] identified machinery accidents as the leading mechanism of injury (34%). Goldcamp et al. [13] stated that the most prevalent causes of farm-related youth deaths were machine related (25%), motor vehicles (17%), and drowning (16%). In our study group, 56% of the injuries resulted from machinery accidents. Tractor-related injuries made another big amount of farm injuries (34%). The reasons for tractor-related injuries are unauthorized drivers at all ages, using the tractor outside the scope of its purpose like transportation, and so forth. The machinery accidents are usually high-energy traumas and cause complex injuries like open fractures. Tractor accidents were evaluated in Aegean region, Turkey, from 2000 to 2005 over 250 farmers [14]. 

In the study group, 90% of the farmers who had tractor-related farm accidents were from an age group of 20 to 50 years old, only 2% younger than 20 and 8% older than 50 years old [14]. Our results also supported almost the same age distribution (20–60: 78%). This data can be explained on the basis of traditions, where very young and very elderly people usually do not participate in heavy farmyard work or transportation. Both young and elderly members of the family may be involved in farmyard accidents, because it is usually a family business [15]. Focusing on the age, elderly (>55 year) workers are especially at risk of fatal injuries and are also over represented in nonfatal injuries [6, 8]. 

The injury-related mortality rate for farmers increases steeply after the age of 60 [3]. In our series, we had 5 patients whose age is over 60, and all the injuries were nonfatal injuries. Although we had many elderly farmers in our region, relatives who are working at urban areas usually come for help to their parents at summer vacations while harvesting, and this may explain our relatively low percentage of elderly people having farmyard accidents. 

Hazardous work-related injuries can affect all ages of farm children even if they are not involved in the work activities themselves [16]. Childhood agricultural injuries represent approximately 19% of all these fatalities and hospitalizations [17]. In our study group, 5 of 41 were under age 20, none of them were fatal, and all of them were boys (data on all fatalities at scene are not collected and discussed). Boys were at a significantly higher risk of exposure compared to girls, because boys engage more frequently in risky behavior [18]. Also, in the adult group, the majority of farmyard injuries were experienced by males [6, 18, 19]. In our study group, 40 of the 41 of the farm injury victims were men, one farm-injured woman has fallen from a tractor. Farmyard injuries in young adults and the elderly usually result from machinery accidents, whereas children tend to be injured by runovers and motor vehicle collisions [5]. 

Open fractures are associated with an increased risk of infection and healing complications [5]. Hartling et al. [17] stated that open wounds to an upper limb were the most common reason for admission to a hospital. In a study by Hansen [20], 45% of the injuries involved the upper extremity, and 45% of these injuries were traumatic amputations and lacerations. In our study group, 9 were upper-extremity injuries, 5 of 6 traumatic amputations were upper extremity amputations, and all these injuries were the result of machinery accidents. More than half of the injuries in our study group were open injuries, and 2 extremities had to be amputated because of the persisting infection and tissue necrosis. The rate of amputations were found to be high (8/41) in our study group however, already 27% of the injuries were crush type on application which once again showing that our series consisted of advanced cases. 

In spite of technological advances in wound management, wound infection has been regarded as the most common reason for nosocomial infection. Infection in a wound delays healing, causes wound breakdown, and increases trauma care and treatment costs [21]. The occurrence of infection in farm injuries was associated with prolonged hospitalization for parenteral antibiotic therapy, multiple surgical debridements, and permanent disability [22]. 

Mean hospitalization time was quite long (24 days) in our study group because of the high incidence of open fractures needing repetitive debridements. Although the attempt was to manage the infection, long hospitalization time can be a reason for secondary infections. Also, the microbiologic load of the farm injuries is different from other industrial injuries. In a study by Agger, initial cultures revealed bacterial growth in 89% of the agricultural wounds and in 63% of the factory wounds [23]. 

Due to the time-dependent nature of the farming activities like harvesting, farmers may work for long hours causing fatigue and carelessness which may lead to serious accidents [24]. There may be a seasonal dispersion pattern of the farm injuries depending on the harvesting time. Richter et al. [24] noticed in their study that January and February are the slow months for farmers. Goldcamp et al. [13] noticed in their study that over 40% of all fatalities occurred in the months June through August. In our study group, most of the injuries were in summer. The seasonal data suggest that both the spring and the early fall are the appropriate times for educational campaigns to prevent farm injuries [25]. Mass media (newspaper, radio, and television) has a responsibility for injury prevention [26]. Education is aimed at persuading the at-risk individuals to change their behaviors. Also, first-aid education may decrease the morbidity of farm injuries. Farm injuries result in considerable physical and emotional disability. All these injuries result in significant work impairment [27]. Since the incidence of farmyard injuries has risen, increased awareness and preventive measures need to be implanted to alter the incidence of accidental injury on the farm [28]. Most of these accidents could be prevented with the use of protective clothing, better education, and safety precautions [4]. 

Most farm accidents and fatalities involve machinery. Proper machine guarding and equipment maintenance can help prevent accidents. An accident-prevention strategy must take into consideration issues regarding the high-risk times [5]. In our study group, we have found a high percentage of amputations and open fractures which are prone to long-time morbidity. It can be easily concluded that high impact of farm accidents ends with physical and emotional disability. Another important effect on society is the age distribution of the patients. Most of the patients (75%) were from an age group of active period, which shows the economical burden of farmyard injury as well as the physical impacts.

## 5. Conclusions 

Farm injuries are more common in summer that resources should be allocated accordingly. Due to the high microbiological load and high incidence of crush-type injuries, repetitive debridements and long duration of hospital stay were needed. Hospitalization time should be minimized as much as possible, in order to limit secondary infections. This can be managed by aggressive initial debridement to reduce hospital stay and reduce the risk of secondary infection. Because of the high seasonal dispersion pattern of the accidents (a summer peak and winter trough), injury prevention programs and hospital major incident planning should be started. Depending on the geographical and seasonal injury data, education campaigns to increase awareness to the hazardous effects of farmyard injuries may be successful to decrease farm-related injuries. First-aid educations and getting prepared for emergency situations can be helpful to minimize the detrimental effects of farm injuries. 

## Figures and Tables

**Table 1:** The anatomical localizations of the injured extremities involved on application.

	İnjury localization	Number
Upper extremity	Hand	9
	Hand and forearm	1
	Forearm	1
	Elbow	1
	Humerus	2
	Upper extremity amputation	5

Lower extremity	Foot	7
	Ankle	1
	Foot and ankle	1
	Crus	6
	Femur	1
	Crus plus femur	1
	Femur plus patella	1
	Extensive soft tissue	1
	Amputation lower extremity	1

Combined upper and lower extremity	Forearm plus crus	2

**Table 2:** The distribution of the etiologic factors and mean age-etiology relationship of the farm injured patients.

	Number	Mean age
Tractor dependent	14	40.7
Wood cutter machine	5	48.4
Farm machine	18	36.6
Fall in the farm	2	53.5
Tree overturn	2	43.5

Total	**41**	**41.9**

**Table 3:** Seasonal dispersion pattern of the farm injuries.

Season	Number of cases
Winter	4
Autumn	11
Spring	11
Summer	15

Total	41

**Table 4:** Incubated cultures and treatment procedures of the infected patients.

Patient	1st culture	Antibiotherapy	2nd culture	Antibiotherapy	3rd culture	Antibiotherapy	Discharge
1	MRSA	Meropenem plus ciprofloxacin (21 days)					
2	—	Cephazolin sodium plus Gentamycin	Klebsiella plus Enterobacter auriginosa (10th day)	Cephazolin sodium plus ciprofloxacin	Enterobacter auriginosa	Imipenem (21 days)	Oral ciprofloxacin
3	Acinetobacter	Imipenem plus Netilmycin	Acinetobacter plus Enterococcus faecalis (10th day)	Imipenem plus netilmicin (21 days)			
4	Acinetobacter	Tazocin plus amikacin/Imipenem plus Netilmicin (21 days)					
5	Enterobacter	Tazocin (14 days)	Acinetobacter plus Pseudomonas (3rd day)	Amikacin (12 days)			ciprofloxacin
6	Enterobacter cloacicae	Tazocin (21 days)					ciprofloxacin
7	Acinetobacter	Ampicillin plus sulbactam (11 days)	Pseudomonas (14th day)	Imipenem (21 days)			
8	Enterococcus	Amoxicillin plus clavulanic acid					
